# Relationship of serum bilirubin concentration to kidney function and 24-hour urine protein in Korean adults

**DOI:** 10.1186/1471-2369-12-29

**Published:** 2011-06-28

**Authors:** Ho Sik Shin, Yeon Soon Jung, Hark Rim

**Affiliations:** 1 Department of Internal Medicine, Kosin University College of Medicine, Busan, Republic of Korea

**Keywords:** Proteinuria, Bilirubin, Glomerular filtration rate

## Abstract

**Background:**

The relationships among serum bilirubin concentration, kidney function and proteinuria have yet to be fully elucidated, nor have these relationships been investigated in Korean adults.

**Method:**

We retrospectively reviewed the medical records of Korean adults who were evaluated at Kosin University Gospel Hospital (Busan, Republic of Korea) during a five-year period from January 2005 to December 2009. We evaluated the relationships among serum bilirubin concentration, estimated glomerular filtration rate (eGFR) and 24-hour urinary protein excretion in a sample of 1363 Korean adults aged 18 years or older.

**Results:**

The values of eGFR <60 mL/min/1.73 m^2 ^and 24-hour urine albumin ≥150 mg/day were observed in 26.1% (n = 356) and 40.5% (n = 553) of subjects, respectively. Fasting glucose levels ≥126 mg/dL were observed in 44.9% (n = 612) of the total sample. After adjustment for potential confounding factors including demographic characteristics, comorbidities and other laboratory measures, total serum bilirubin was positively associated with eGFR and negatively associated with proteinuria both in the whole cohort and in a subgroup of diabetic individuals.

**Conclusions:**

To our knowledge, this is the first hospital-based study specifically aimed at examining the relationships among serum total bilirubin concentration, 24-hour urine protein and kidney function in Korean adults. We demonstrated that serum total bilirubin concentration was negatively correlated with 24-hour urine protein and positively correlated with eGFR in Korean non-diabetic and diabetic adults.

## Background

The prevalence of chronic kidney disease is increasing worldwide [1,2]. Reduced eGFR and abnormal proteinuria have been associated with increased risk of end stage renal disease (ESRD), cardiovascular disease and other comorbidities [3-6]. Because renal disease often progresses to ESRD, the identification of risk factors for kidney disease progression is essential.

Increased concentrations of serum bilirubin have long been used as a marker of liver dysfunction. In addition, serum bilirubin is not merely an end product of heme degradation but is also a potent antioxidant that acts via inhibitions of NADPH oxidase, a key source of oxidants in phagocytic and non-phagocytic cells, and of protein kinase C activity [7-9].

Information on the associations of serum bilirubin concentration with renal function and proteinuria is limited and controversial. Fukui et al. found that total serum bilirubin was positively associated with eGFR and negatively associated with albuminuria in a hospital-based sample of 633 Japanese type 2 diabetic patients [10], indicating that bilirubin has a potential renoprotective effect. In contrast, Targher et al. found that a higher total serum bilirubin was significantly associated with lower eGFR in both non-diabetic and diabetic individuals in a group of 2678 unselected outpatients 35 years of age or older [11].

To date, the associations of serum bilirubin concentration with kidney function and proteinuria have not been established in a Korean adult population. Therefore, we examined the associations among serum bilirubin concentration, eGFR and the degree of 24-hour urinary protein excretion in patients at Kosin University Gospel Hospital (Busan, Republic of Korea) during a five-year period from January 2005 to December 2009.

## Methods

### Patients

We retrospectively reviewed the medical records of Korean adults patients who had visited Kosin University Gospel Hospital (Busan, Republic of Korea) from January 2005 to December 2009 and evaluated the relationships among serum bilirubin concentration, eGFR and 24-hour urinary protein excretion in a sample of 1363 Korean adults aged 18 years or older (669 men, 694 women, aged 54.6 ± 15.1 years, range 18-93 years). The study was reviewed and approved by the Ethics Committee of Kosin University Gospel Hospital.

### Clinical and biochemical assessments

Type 2 diabetes was diagnosed according to the Report of the Expert Committee on the Diagnosis and Classification of Diabetes Mellitus [12]. Nephropathy was graded as follows: normoproteinuria, urinary protein excretion less than 150 mg/day and macroproteinuria, greater than 150 mg/day.

As a surrogate for measuring renal function in the subjects, we estimated the GFR using a simplified form of the Modification of Renal Disease (MDRD) equation [13]. The equation is as follows:

The exclusion criteria were as follows:

1) patients with malignant disease, liver cirrhosis, or hematologic disease;

2) patients with advanced renal dysfunction (serum creatinine level higher than 2.0 mg/dL);

3) patients whose serum bilirubin concentrations were greater than 1.2 mg/dL due to the high possibility of Gilbert syndrome.

4) patients with chronic hepatitis B or C

Total serum bilirubin concentrations (normal ranges: 0.2-1.0 mg/dL) were measured using an enzymatic method with bilirubin oxidase on an automatic analyzer (Hitachi 7600). Serum total cholesterol, high-density lipoprotein cholesterol, and triglyceride concentrations were assessed using standard enzymatic methods. Twenty-four-hour urine samples were collected from patients who demonstrated proteinuria in a dipstick urine test.

### Statistical analysis

The results are presented as the mean ± SD. We used Student's *t *test to compare the means and Pearson's correlation coefficient to evaluate the relationships between parameters. The association between serum total bilirubin and eGFR and the association between serum total bilirubin and 24 hour urine protein were investigated using linear (adjusted) regression models in the whole group and in subgroups of patients stratified by diabetes status. The covariates included in adjusted regression models were age, sex, diabetes, hypertension, total cholesterol, LDL-cholesterol, HDL-cholesterol and triglyceride. The results were considered significant when the *P *value was less than 0.05. All statistical analyses were performed using the Statistical Package for the Social Sciences (SPSS) version 12.0 (SPSS Inc., Chicago, IL, USA).

## Results

Characteristics of the 1363 subjects enrolled in this study are shown in Table 1.

**Table 1:** Clinical characteristics of subjects

Variable	**Mean ± s.d**.
**n**	1,363
**Age (years)**	55.6 ± 14.1
**Sex,%**	
Males	49.1
Females	50.9
**Diabetes,%**	44.9
**Hypertension,%**	14.0
**Measurements**	
Urine protein, mg/day	806 ± 2,150
WBC, mm^3^	7,619 ± 3,083
Hb, g/dL	12.2 ± 1.9
Platelet, mm^3^	260,398 ± 98,162
Protein, g/dL	6.7 ± 2.7
Albumin, g/dL	3.8 ± 0.6
Total cholesterol, mg/dL	204 ± 77
LDL Cholesterol, mg/dL	115 ± 43
HDL cholesterol, mg/dL	44 ± 15
Triglycerides, mg/dL	158 ± 209
AST, IU/L	28 ± 22
ALT, IU/L	27 ± 24
Fasting blood glucose, mg/dL	144 ± 73
BUN, mg/dL	18.6 ± 10.4
Creatinine, mg/dL	1.16 ± 0.64
eGFR, mL/min/1.73 m^2^	75 ± 31
Uric acid, IU/L	5.4 ± 2.0
Sodium, mEq/L	140.6 ± 4.8
Potassium, mEq/L	4.2 ± 0.6
Calcium, mg/dL	9.1 ± 0.7
Phosphorus, mg/dL	4.0 ± 3.1
Chloride, mEq/L	102 ± 11
total CO_2_, mEq/L	26.4 ± 4.9
Total Bilirubin, mg/dL	0.75 ± 0.21
Direct Bilirubin, mg/dL	0.30 ± 0.09

Among the 1363 adult subjects, the mean age and total bilirubin were 55.6 (± 14.1) years and 0.75 (± 0.21) mg/dL, respectively. An eGFR <60 mL/min/1.73 m^2 ^and a 24-hour urine protein ≥150 mg/day were present in 26.1% (n = 356) and 40.5% (n = 553) of the subjects, respectively. Diabetes and hypertension were present in 44.9% (n = 612) and 14.0% (n = 191) of patients.

**Figure 1:**
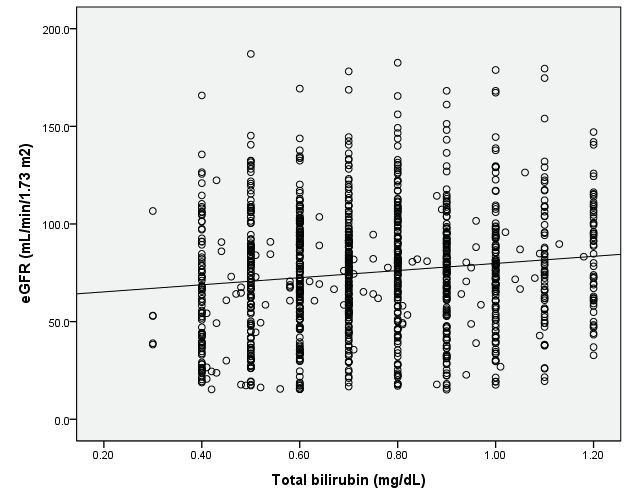
**Correlation between serum total bilirubin concentration and eGFR in whole subjects (r = 0.128, p = 0.0001)**.

**Figure 2:**
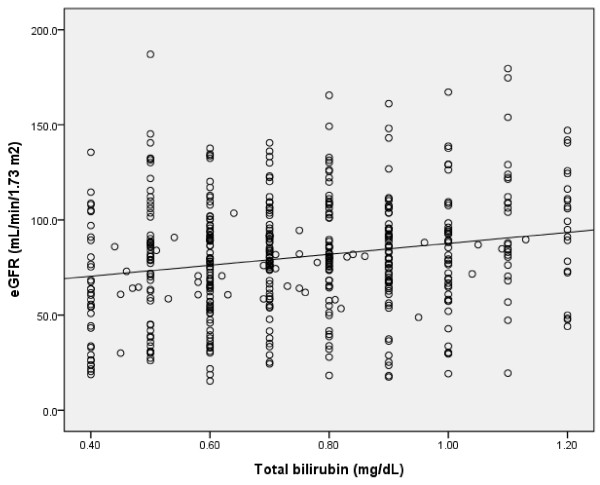
**Correlation between serum total bilirubin concentration and eGFR in DM subjects (r = 0.202, p = 0.0001)**.

**Figure 3:**
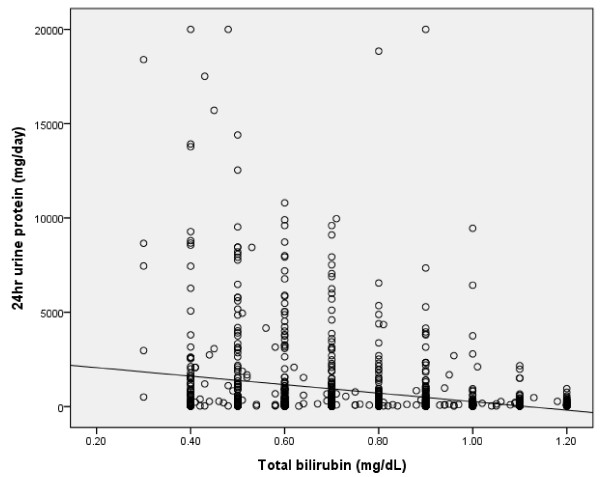
**Correlation between serum total bilirubin concentration and 24 hour urine protein in whole subjects (r = -0.228, p = 0.0001)**.

**Figure 4:**
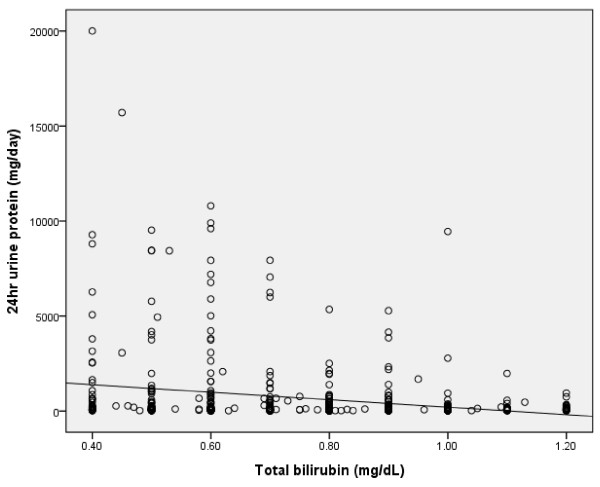
**Correlation between serum total bilirubin concentration and 24 hour urine protein in DM subjects (r = -0.227, p = 0.0001)**.

The clinical and biochemical characteristics of subjects stratified according to eGFR category are summarized in Table 2. Compared with patients with normal or near-normal eGFR, persons with lower eGFR were older, more likely to be male, and had greater prevalence for high serum total bilirubin concentration. A positive correlation was found between serum total bilirubin concentration and eGFR in all subjects and in diabetic patients (r = 0.128, p = 0.0001; r = 0.202, p = 0.0001) (Figure 1, 2). An inverse correlation was found between total serum bilirubin concentration and 24-hour urine albumin in all subjects and in subjects with diabetes (r = -0.228, p = 0.0001; r = -0.227, p = 0.0001) (Figure 3, 4).

**Table 2:** Age-gender standardized baseline demographics and laboratory results accordings to eGFR in the entire cohort (n = 1,363)

eGFR Characteristics	**≥90 mL/min/1.73 m**^**2**^**, n = 564**	**60-89 mL/min/1.73 m**^**2**^**, n = 443**	**30-59 mL/min/1.73 m**^**2**^**, n = 265**	**15-29 mL/min/1.73 m**^**2**^**, n = 91**	p-value
**Age, years**	50.1 ± 13.8	57.3 ± 13.8	59.3 ± 13.7	59.4 ± 14.6	0.0001
T^1)^	a	b, c	c	c	
**Sex,%**					
Males	46	48.3	54.3	56	
Females	54	51.7	45.7	44	
**Diabetes,%**	52.8	45.1	32.8	29.4	
**Hypertension,%**	10.1	12.1	23.3	21.2	
**Measurements**					
Urine protein, mg/day	572 ± 1,871	744 ± 2,366	1,231 ± 2,236	2,050 ± 3047	0.0001
T^1)^	a	a, b	b	c	
WBC, mm^3^	7,420 ± 3,116	7,534 ± 3,112	7,940 ± 3,003	7,909 ± 2,967	0.252
Hb, g/dL	12.5 ± 1.7	12.5 ± 1.7	11.6 ± 1.9	10.8 ± 1.8	0.0001
T^1)^	a	a	b	c	
Platelet, mm^3^	276,222 ± 103,955	259,255 ± 92,563	250,831 ± 96,831	235,755 ± 87,964	0.002
T^1)^	a	a, b	a, b	b	
Protein, g/dL	6.7 ± 3.3	6.6 ± 0.9	6.7 ± 3.8	6.3 ± 0.8	0.447
Albumin, g/dL	3.8 ± 0.7	3.8 ± 0.6	3.6 ± 0.7	3.5 ± 0.6	0.0001
T^1)^	a	a	a, b	b	
Total cholesterol, mg/dL	204 ± 93	208 ± 71	207 ± 87	205 ± 72	0.215
LDL Cholesterol, mg/dL	126 ± 55	113 ± 44	109 ± 34	112 ± 40	0.103
HDL cholesterol, mg/dL	46 ± 16	45 ± 16	42 ± 14	40 ± 12	0.001
T^1)^	a	a, b	b, c	c	
Triglycerides, mg/dL	170 ± 360	151 ± 127	155 ± 95	180 ± 192	0.657
AST, IU/L	29 ± 27	29 ± 20	27 ± 16	29 ± 38	0.108
ALT, IU/L	28 ± 25	28 ± 23	24 ± 20	26 ± 47	0.197
Fasting blood glucose, mg/dL	153 ± 81	142 ± 74	127 ± 62	127 ± 69	0.0001
T^1)^	a	a, b	b	b	
BUN, mg/dL	12.5 ± 4.5	16.1 ± 5.6	24.3 ± 9.4	38.1 ± 15.5	0.0001
T^1)^	a	a	b	c	
Creatinine, mg/dL	0.71 ± 0.13	0.96 ± 0.16	1.51 ± 0.33	2.86 ± 0.69	0.0001
T^1)^	a	b	c	d	
Total Bilirubin, mg/dL	0.77 ± 0.22	0.76 ± 0.21	0.72 ± 0.22	0.67 ± 0.21	0.0001
T^1)^	a, b	a, b	b, c	c	
Direct Bilirubin, mg/dL	0.31 ± 0.09	0.30 ± 0.09	0.30 ± 0.10	0.28 ± 0.09	0.222

1) The same letters indicate non-significant difference between groups based on Turey's multiple comparison test

When participants were stratified into categories based on proteinuria (Table 3), persons with higher proteinuria were hypoalbuminemic, more likely to be male, hypercholesterolemic (although level of LDL was similar) and had lower values of total bilirubin compared with those of patients with normal proteinuria.

**Table 3:** Age-gender standardized baseline demographics and laboratory results according to 24 urine protein excretion in the entire cohort (n = 1,363)

24 hour urine protein (mg/day)	<150	≥ 150	P value
**Characteristics**	n = 810	n = 553	
**Age, years**	55.9 ± 13.9	55.9 ± 14.9	0.642
**Sex,%**			0.0001
Males	44.7	55.5	
Females	55.3	44.5	
**Diabetes,%**	47.4	41.2	0.027
**Hypertension,%**	8.7	21.6	0.0001
**eGFR,%, mL/min/1.73 m**^**2**^			0.0001
≥90	35.8	23.1	
60-89	44.4	31.1	
30-59	16.6	31.5	
15-29	3.2	14.3	
**Measurements**			
WBC, mm^3^	7,296 ± 2,940	8,044 ± 3,216	0.0001
Hb, g/dL	12.5 ± 1.7	11.8 ± 1.9	0.0001
Platelet, mm^3^	257,821 ± 93,576	263,793 ± 103,896	0.315
Protein, g/dL	6.8 ± 1.6	6.5 ± 3.7	0.097
Albumin, g/dL	4.0 ± 0.4	3.5 ± 0.7	0.0001
Total cholesterol, mg/dL	194 ± 49	219 ±103	0.0001
LDL Cholesterol, mg/dL	115 ± 37	115 ± 49	0.952
HDL cholesterol, mg/dL	45 ± 15	43 ± 16	0.023
Triglycerides, mg/dL	148 ± 151	174 ± 272	0.037
AST, IU/L	27 ± 20	29 ± 25	0.139
ALT, IU/L	27 ± 21	27 ± 28	0.898
BUN, mg/dL	16.3 ± 7.9	21.7 ± 12.4	0.0001
Creatinine, mg/dL	0.99 ± 0.41	1.39 ± 0.79	0.0001
Total Bilirubin, mg/dL	0.78 ± 0.20	0.71 ± 0.22	0.0001
Direct Bilirubin, mg/dL	0.30 ± 0.09	0.29 ± 0.09	0.003

When participants were stratified into categories based on fasting blood glucose (Table 4), diabetic patients were hypoalbuminuric compared with non-diabetic patients. No significant differences were found with regard to serum total bilirubin concentration between participants with normal and abnormal fasting blood glucose levels.

**Table 4:** Age-gender standardized baseline demographics and laboratory results according to fasting blood glucose (n = 1,363)

Fasting blood glucose, mg/dL	<126	≥ 126	P value
**Characteristics**			
**Age, years**	53.9 ± 14.6	57.7 ± 13.1	0.0001
**Sex,%**			0.414
Males	48.1	50.3	
Females	51.9	49.7	
**eGFR,%, mL/min/1.73 m**^**2**^			0.0001
≥90	27.5	34.1	
60-89	36.3	41.9	
30-59	26.6	18.2	
15-29	9.6	5.8	
**Measurements**			
24 hour urine protein, mg/day	913 ± 2,350	676 ± 1,870	0.038
WBC, mm^3^	7,368 ± 2,942	7,965 ± 3,239	0.001
Hb, g/dL	12.0 ± 1.8	12.3 ± 1.8	0.003
Platelet, mm^3^	258,479 ± 97,902	263,037 ± 98,560	0.438
Protein, g/dL	6.7 ± 3.2	6.6 ± 1.9	0.639
Albumin, g/dL	3.7 ±0.7	3.8 ± 0.5	0.046
Total cholesterol, mg/dL	207 ± 78	201 ± 75	0.211
LDL Cholesterol, mg/dL	114 ± 47	115 ± 38	0.877
HDL cholesterol, mg/dL	46 ± 16	42 ± 14	0.0001
Triglycerides, mg/dL	140 ± 97	179 ± 286	0.002
AST, IU/L	28 ± 20	28 ± 25	0.861
ALT, IU/L	24 ± 19	30 ± 29	0.0001
BUN, mg/dL	19.0 ± 11.0	18.1 ± 9.6	0.16
Creatinine, mg/dL	1.24 ± 0.69	1.06 ± 0.55	0.0001
Total Bilirubin, mg/dL	0.75 ± 0.21	0.76 ± 0.21	0.499
Direct Bilirubin, mg/dL	0.30 ± 0.09	0.30 ± 0.08	0.705
eGFR, mL/min/1.73 m^2^	71 ± 31	80 ± 30	0.0001

## Discussion

To our knowledge, this is the first hospital-based study specifically aimed at examining the associations among serum total bilirubin concentration, 24-hour urine protein and kidney function in Korean adults.

We retrospectively reviewed the medical records of 1363 adults aged 18 years or older who were seen at Kosin University Gospel Hospital (Busan, Republic of Korea) in the five-year period from January 2005 to December 2009. This study demonstrated that serum total bilirubin concentration was negatively correlated with 24-hour urine protein and was positively correlated with eGFR in Korean non-diabetic and diabetic adults.

Our findings are in contrast to the results of Targher et al. [11] in their observational large hospital-based sample of 2678 adult outpatients (mean age: 55 ± 18 years; 43% male), including 210 diabetic patients. In that study, they found that serum total bilirubin was inversely associated with eGFR in both non-diabetic (r = -0.17; p < 0.0001) and diabetic patients (r = -0.14; p < 0.05). However, no information was available on albuminuria, comorbidites, alcohol consumption or other important potential confounders [11]. In this study, we could not analyzed the effects of alcohol and smoking on proteinuria, because we were unable to locate data on alcohol drinking and smoking.

The significant associations between total serum bilirubin and eGFR or albuminuria observed in diabetic individuals of our cohort were the same as those from Fukui et al. [10]. That previous study found that total serum bilirubin was positively associated with eGFR and negatively associated with albuminuria in a hospital-based sample of 633 Japanese type 2 diabetic patients (mean age: 64.4 ± 11.5 years; 52% male). However, in that study, no adjustment was made for important confounders, such as eGFR. In our study, a positive correlation was found between total serum bilirubin concentration and eGFR in all subjects as well as in diabetic patients.

Based on *in vitro *as well as animal studies, bilirubin is generally recognized as an important antioxidant substance. Kumar et al. demonstrated that serum bilirubin concentration is inversely correlated with a marker of oxidative stress and is positively correlated with antioxidative enzyme activities such as those of superoxide dismutase, catalase, and glutathione peroxidase [14]. These results are also supported by clinical studies focused on the protective effects of serum bilirubin concentration on atherosclerosis [15-17]. In this study, serum bilirubin concentration correlated negatively with proteinuria and positively correlated with renal function, and total cholesterol in the normal proteinuria group level was lower than that in the abnormal proteinuria group. However, we were unable to assess the antioxidant effect of serum bilirubin, because tests for an oxidative stress marker and antioxidant enzyme were not carried out.

In addition to being an antioxidant, bilirubin also has anticomplement properties that protect against inflammation [18]. Furthermore, bilirubin has been suggested to have cytoprotective properties through its influence on protein kinase C [19]. Through these mechanisms, bilirubin could protect diabetic patients from the development and progression of diabetic nephropathy [20-23]. However, in this study, the 24-hour proteinuria level in the diabetic group was lower than that in the non-diabetic group because CKD patients were included in the non-diabetic group.

In results of this study, we might think serum bilirubin concentration may be utilized as a provisional new risk factor of diabetic nephropathy that can be measured easily in the clinical laboratory and applied in medical practice.

It could be hypothesized that the bilirubin-kidney dysfunction relationship primarily reflects the association of serum bilirubin concentration with non-alcoholic fatty liver disease (NAFLD). NAFLD represents the most common cause of mild to moderate increases in serum bilirubin and other liver enzymes in Western countries [24]. Recent studies have found that NAFLD is independently associated with an increased incidence of CKD in both non-diabetic and diabetic populations [25,26].

Limitations of our study include its cross-sectional design, which allows us to identify only associations and should not yield any conclusions about causation. In addition, results of this study may not be applicable to the general population or to patients with type 2 diabetes in a primary care clinic because the data were collected from patients in an outpatient clinic of a university hospital. This study's results are not definitive because a Pearson's correlation coefficient of less than ± 0.25 is generally considered to be a weak correlation. We think prompt further research into this interesting correlation will be needed. Second, we were unable to explain the reason why both the non-diabetic and diabetic group had the same results. Third, liver ultrasonography for diagnosing NAFLD was not performed. Finally, we used eGFR instead of a directly measured GFR to assess renal function. It is known that current eGFR experiences greater inaccuracy in populations with no known CKD than in those with CKD. Nonetheless, the current eGFR technique facilitates the detection, evaluation, and management of renal disease.

To our knowledge, this is the first study to investigate the relationship between serum bilirubin concentration and 24-hour urine protein excretion in non-diabetic subjects and in patients with type 2 diabetes. Although we are unable to determine whether hypobilirubinemia has a causative effect, these findings suggest that hypobilirubinemia combined with diabetes might be associated with advanced diabetic nephropathy. Large prospective trials are needed to better assess the effects of bilirubin on diabetic nephropathy in patients with type 2 diabetes.

## Conclusions

our findings suggest that increasing serum total bilirubin concentration is associated with increasing eGFR and decreasing albuminuria in Korean non-diabetic and diabetic adults.

## Competing interests

The authors declare that they have no competing interests.

## Authors' contributions

HSS, M.D. and HR, M.D. participated in the design of the study and performed the statistical analysis. YSJ, M.D. conceived of the study, and participated in its design and coordination. All authors read and approved the final manuscript.

## Pre-publication history

The pre-publication history for this paper can be accessed here:

http://www.biomedcentral.com/1471-2369/12/29/prepub
